# Quantum-limited heat conduction over macroscopic distances

**DOI:** 10.1038/nphys3642

**Published:** 2016-02-01

**Authors:** Matti Partanen, Kuan Yen Tan, Joonas Govenius, Russell E. Lake, Miika K. Mäkelä, Tuomo Tanttu, Mikko Möttönen

**Affiliations:** QCD Labs, COMP Centre of Excellence, Department of Applied Physics, Aalto University, P.O. Box 13500, FI–00076 Aalto, Finland

## Abstract

The emerging quantum technological apparatuses^1^, ^2^, such as the quantum computer^3^–^6^, call for extreme performance in thermal engineering^7^. Cold distant heat sinks are needed for the quantized electric degrees of freedom due to the increasing packaging density and heat dissipation. Importantly, quantum mechanics sets a fundamental upper limit for the flow of information and heat, which is quantified by the quantum of thermal conductance^8^–^10^. However, the short distance between the heat-exchanging bodies in the previous experiments^11^–^14^ hinders their applicability in quantum technology. Here, we present experimental observations of quantum-limited heat conduction over macroscopic distances extending to a metre. We achieved this improvement of four orders of magnitude in the distance by utilizing microwave photons travelling in superconducting transmission lines. Thus, it seems that quantum-limited heat conduction has no fundamental distance cutoff. This work establishes the integration of normal-metal components into the framework of circuit quantum electrodynamics^15^–^17^ which provides a basis for the superconducting quantum computer^18^–^21^. Especially, our results facilitate remote cooling of nanoelectronic devices using far-away in-situ-tunable heat sinks^22^, ^23^. Furthermore, quantum-limited heat conduction is important in contemporary thermodynamics^24^, ^25^. Here, the long distance may lead to ultimately efficient mesoscopic heat engines with promising practical applications^26^.

The quantum of thermal conductance, $G_{\text{Q}}=\frac{\pi k_{B}^{2}T}{6\hbar }$, provides the fundamental upper limit for heat conduction through a single channel^8^, ^9^. Here, *T* is the temperature, *k_B_* denotes the Boltzmann constant, and *ħ* is the reduced Planck’s constant. This limit applies to fermions and bosons as the heat carriers as well as to so-called anyons obeying even more general statistics^9^. Although a few observations of quantum-limited heat conduction have been reported, the studied distances were shorter than 100 *μ*m in all previous experiments: phononic heat conduction through four parallel submicrometre dielectric wires each supporting four vibrational modes^11^, electromagnetic heat conduction in a superconducting loop over a 50-*μ*m distance^12^, ^13^, and electronic heat conduction through an extremely short quantum point contact engineered in a two-dimensional electron gas^14^.

Achieving the quantum limit is challenging since it requires ballistic transport of the heat carriers. For instance, ballistic transport of electrons is not feasible over long distances in normal metals due to scattering with phonons, other electrons, and lattice defects. Photons, unlike many other carriers of heat, can travel macroscopic distances without significant scattering, for example, in optical fibres or superconducting waveguides. Thus, photons seem ideal for long-distance thermal engineering and provide attractive opportunities for various quantum thermodynamics experiments^24^. To our knowledge, however, itinerant photons have not been previously employed in experimental studies of the quantum of thermal conductance.

In this Letter, we experimentally study quantum-limited heat conduction through a single channel formed by photons travelling in a long superconducting waveguide in a single transverse mode. Since this heat transport does not directly depend on the temperature of the substrate phonons, it provides an efficient method for remote temperature control. The superconducting waveguide is terminated at both ends by resistors composed of mesoscopic normal-metal islands (Islands A and B in Fig. 1). We measure the temperatures of the Islands A and B, and vary the temperature of Island B. A characteristic signal of photonic heat transport in our experiments is the increasing response of the temperature of Island A to the controlled temperature changes of Island B with decreasing phonon bath temperature (Figs. 2 and 3). The measured temperatures well agree with the thermal model, implying that the heat conduction essentially reaches the quantum limit. These observations constitute our main evidence of quantum-limited heat conduction over macroscopic distances.

Figure 1 shows the structure of the sample used in the experiments together with the measurement scheme. We study several samples with different parameters as presented in Table 1. The length of the coplanar waveguide is either 20 cm or 1 m, and it has a double-spiral structure on a silicon chip with the size of 1 × 1 cm^2^ or 2 × 2 cm^2^, respectively. In all samples, the normal-metal islands terminating the waveguide have two galvanic contacts to superconducting lines: one to the centre conductor of the waveguide and the other to the ground plane. In the control sample, the centre conductor is shunted to ground to extinguish photonic heat conduction (Supplementary Fig. 3).

There are four nominally identical normal-metal-insulator-superconductor (NIS) tunnel junctions at each island. The voltage-biased (*V*_B_) pair of NIS junctions at Island B is used to control the normal-metal electron temperature *T*_B_ as shown in Fig. 1i, whereas the current-biased (*I*_th,A_, *I*_th,B_) pair at each island is used for measuring the electron temperature (*T*_A_, *T*_B_) (Methods). We mainly focus on Sample A1 which exhibits the highest photonic thermal conductance. It also achieves the lowest electron temperature, below 90 mK at the 10-mK base temperature of the cryostat. The other samples show higher minimum electron temperatures, which increases the uncertainty in their thermometry at the very low temperatures.

We analyse the thermal conductance between the Islands A and B, *G*_AB_, and those to the phonon bath, *G*_A0_ and *G*_B0_, as schematically presented in Fig. 1d. By linearizing the heat flows of Island A at small temperature differences, energy conservation yields a differential temperature response: d*T*_A_/d*T*_B_ = *G*_AB_/(*G*_AB_ + *G*_A0_). If the conductance *G*_AB_ between the islands is quantum limited and the heat conduction to the bath stems from qualitatively different phenomena, *G*_AB_ dominates over *G*_A0_ at low enough temperatures. Thus, the temperature response generally tends to unity with decreasing temperatures.

The photonic net power flow from normal-metal Island A to B is given by^27^ (Methods)

(1) $$P_{\Gamma }=\int_{0}^{\infty }\frac{\text{d}\omega }{2\pi }\hbar \omega |t(\omega ){|}^{2}\left[\frac{1}{\exp\left(\frac{\hbar \omega }{k_{\text{B}}T_{\text{A}}}\right)-1}-\frac{1}{\exp\left(\frac{\hbar \omega }{k_{\text{B}}T_{\text{B}}}\right)-1}\right]$$

where $|t(\omega ){|}^{2}$ is a transmission coefficient that depends on the photon angular frequency *ω*, the characteristic impedance of the transmission line, and the resistances of the terminating normal-metal islands (see equation (9) in Methods for details). If the characteristic impedance of the transmission line equals the island resistances, we have $|t(\omega ){|}^{2}=1$. In this case, an analytical solution is obtained, $P_{\Gamma }=\frac{\pi k_{\text{B}}^{2}}{12\hbar }(T_{\text{A}}^{2}-T_{\text{B}}^{2})$, which can further be expressed in terms of the quantum of thermal conductance as *P*_Γ_ = *G*_Q_(*T*_A_ – *T*_B_), where $G_{\text{Q}}=\frac{\pi k_{B}^{2}T}{6\hbar }$ with *T* = (*T*_A_ + *T*_B_)/2. The thermal conductance to the phonon bath at temperature *T*_0_ can be approximated by the electron–phonon conductance as $G_{\text{A0}}\approx G_{\text{ep},\text{A}}=5{\Sigma {}_{\text{N}}\Omega }_{\text{A}}T_{0}^{4}$ in Island A (Methods). Here, Σ_N_ is a material parameter describing the strength of the electron-phonon coupling in the normal metal, and Ω_A_ is the volume of Island A. Thus, one obtains a simple theoretical prediction without any free parameters based on quantum-limited photonic heat conduction and electron–phonon coupling

(2) $$\frac{\text{d}T_{\text{A}}}{\text{d}T_{\text{B}}}=\frac{1}{1+aT_{0}^{3}}$$

where $a=30\Sigma_{\text{N}}\Omega_{\text{A}}\hbar /(\pi k_{\text{B}}^{2})$ is a predetermined constant. We also devise a full thermal model shown in Supplementary Fig. 2 for a more accurate description of the heat flows (Methods).

Figure 2a–d shows for Sample A1 changes in the island temperatures due to the bias voltage *V*_B_ at various bath temperatures. The experimental observations are in good agreement with the full thermal model. Although the quantum of thermal conductance is equally relevant in the case of heating, we mainly discuss cooling below since here the net power dissipated at the sample *V*_B_*I*_B_ > 0 is small, and hence any parasitic heating of Island A due to this power is weak (Methods). Furthermore, observation of cooling at Island A qualitatively excludes the possibility of phonons to act as the heat carriers since for any finite bias voltage, there is net heat dissipated in the vicinity of the NIS junctions eventually leading to slight heating of the phonon bath. The maximum cooling of Island B is obtained at its bias voltages $V_{\text{B}}≲2\Delta /e\approx 0.4\;\text{mV}$, where Δ is the superconductor energy gap and *e* is the elementary charge. This optimal cooling point arises due to the competition between the increasing magnitude of the tunnelling current with the bias voltage and the change from cooling-inducing tunnelling to heating of the normal metal.

Figure 2e shows maxima in the absolute temperature drops of both islands between 100 and 200 mK. The observed non-monotonic behaviour is explained by the competition between the increasing cooling power of the NIS junctions with increasing temperature and the increasing quasiparticle thermal conductance from the islands eventually to the phonon bath *G*_A/B0_ (Methods). Quasiparticles may also contribute to the heat conduction between the islands, but in ref. 13, the quasiparticle heat conduction over a 50-*μ*m distance was observed to essentially vanish below 200 mK. Due to orders of magnitude longer distances in our samples, we expect even weaker quasiparticle heat conduction. Furthermore, the essentially vanishing temperature response for the control sample together with the increasing ratio of Δ*T*_A_/Δ*T*_B_ with decreasing temperature in Fig. 2f indicate that below 200 mK, the photonic channel starts to dominate the heat conduction between the islands in Sample A1.

To accurately analyse the photonic heat conduction, we show the temperature of Island A in Fig. 3a as a function of the electron temperature of Island B for different bath temperatures. In Sample A1, the curvatures of *T*_A_ as a function of *T*_B_ are negative, which is in stark contrast to the positive curvature observed for the control sample. This fundamental difference is due to the absence of the photonic heat conduction in the control sample. In Samples A2 and A3 (Table 1), the curvatures resemble that of A1 (data not shown). At high bath temperatures, *T*_A_ is almost independent of *T*_B_, which is a consequence of the strong coupling to phonons, i.e., *G*_A0_ ≫ *G*_AB_. Figure 3b shows the differential temperature response, d*T*_A_/d*T*_B_, extracted at the lowest *T*_B_ obtained for each bath temperature. The steep increase in d*T*_A_/d*T*_B_ for decreasing bath temperatures is a signature of the photonic heat conduction: the thermal conductance between the islands, *G*_AB_, determined by the photonic heat conduction, dominates over the conductance to the bath, *G*_A0_.

In addition, Fig. 3b shows predictions of the simplified model according to equation (2). Despite its simplicity, it captures the essential features of the experimental data of Samples A1 and A2 which exhibit photonic heat conduction very close to the quantum limit, *G*_Q_. The deviation between the data and the simplified model at high temperatures is due to the neglected quasiparticle heat conduction between the islands and their reservoirs which increases *G*_A0_. At low temperatures, the discrepancy arises from the saturation of the electron temperatures not present in the simplified model. Due to the saturation in the experiments, the heat conductances do not reach their zero-temperature values, and hence d*T*_A_/d*T*_B_ does not tend to unity. These observations bring insight to the good agreement between the experimental observations and the full thermal model.

The inset in Fig. 3b shows the thermal conductance between the islands extracted as a free parameter to match the thermal models with the measured differential temperature response. Using two different literature values^7^ for the electron phonon coupling constant, Σ_N_ = 2 × 10^9^ WK^−5^m^−3^ and 4 × 10^9^ WK^−5^m^−3^, we obtain from the simplified thermal model *η* = *G*_AB_/*G*_Q_ = 0.42 and 0.86, respectively, at *T*_0_ ≈ 150 mK. At zero temperature, *η* vanishes for the simplified model due to the temperature saturation, and above 200 mK the suppressed temperature response limits the accuracy of these estimates. The full thermal model with the parameters shown in Supplementary Table 1 yields *η* = 0.8–1.15 below 200-mK bath temperature.

In summary, we experimentally demonstrated quantum-limited heat conduction over macroscopic distances. The on-chip design of the resistors potentially enables their utilization in a multitude of different applications, including the initialization of quantum bits^22^, ^28^. The methods developed in this study may also be used in the future to implement efficient heat transfer between separate chips and temperature stages of the cryostat. For example, the remotely cooled quantum device may be operated at a typical base temperature whereas the cold reservoir may be located at a lower-temperature stage which is incompatible with the relatively large power consumption of the actual device.

The maximum distance for efficient heat transport is limited in practise by the internal losses in the waveguide. With standard techniques utilized in high-quality resonators (Methods), one may achieve distances beyond 1 km. The temperature response time between the islands arising from a 1-km distance and the speed of light in the waveguide is roughly ten microseconds which should be experimentally observable using rf readout.

## Methods

### Sample fabrication

The samples are fabricated on 0.5-mm-thick silicon wafers with 300-nm-thick thermally grown silicon oxide layers. The transmission lines are fabricated in an optical-lithography process using a mask aligner and an electron beam evaporator. The wafers are cleaned with reactive ion etching before the metal deposition. The Al film has a thickness of 200 nm, on top of which films of Ti and Au are deposited with thicknesses of 3 and 5 nm, respectively, to prevent oxidation.

The nanostructures are fabricated with electron beam lithography. The mask consists of poly(methyl methacrylate) and poly[(methyl methacrylate)-*co*-(methacrylic acid)] layers, which enable a large undercut necessary for three-angle shadow evaporation. Prior to the metal deposition, the samples are cleaned with argon plasma in the electron beam evaporator. As the first metal, we deposit an Al layer which is oxidized *in situ* introducing the insulator layer for the NIS junctions. Subsequently, a layer of normal metal is deposited followed by a layer of Al. The normal metal is either AuPd (mass ratio 3:1) or Cu. Lift-off of the excess metal is performed with acetone followed by cleaning with isopropanol.

### Measurements

The electrical measurements are performed at millikelvin temperatures achieved with a commercial cryogen-free dilution refrigerator. The phonon temperature is controlled by applying a constant heating power at a resistive mixing chamber heater, after which we wait until a steady state is reached.

The chip is attached to a sample holder containing a printed circuit board (PCB), to which the sample is connected by Al bond wires. The PCB is connected to room-temperature measurement setup with lossy coaxial cables. To suppress electrical noise, the power-line-powered devices are connected to the sample through opto-isolators. Battery-powered amplifiers and voltage and current sources are connected to the sample without opto-isolation. The voltage *V*_B_ is swept slowly (down to 1 *μ*V/s) to avoid apparent hysteresis. Furthermore, the measurements are repeated several times, and the data points with clear disturbance from random external fluctuations are excluded.

The minimum electron temperature of the islands is higher than the base temperature as a consequence of noise and high-temperature radiation leaking to the sample through the measurement lines and holes in the radiation shields. As described below, this small heating power is taken into account in the full thermal model. The current sources for the island thermometers were different for some samples and produced less noise for Sample A1 than for the other samples, which reduced the resulting electron temperature. In addition, smaller thermometer bias currents were sufficient in Sample A1 due to the lower noise level of the measured signal, thus enabling the observation of lower electron temperatures. The minimum temperatures may be further reduced by other technical improvements such as improved shielding and filtering. However, the electron temperatures achieved in this work are sufficient for the observation of quantum-limited heat conduction over macroscopic distances.

### Photonic heat conduction

Here, we derive equation (1) for the photonic heat conduction starting from the first-principles circuit quantum electrodynamics. Previously, our case of two islands coupled with a transmission line has been studied with the help of classical circuit theory^27^. These results can also be obtained using path integrals^25^. In contrast, we analyse the system using methods discussed in ref. 29. In particular, a terminating resistor is treated as a semi-infinite transmission line with a characteristic impedance equal to its resistance. The photon annihilation operators are defined using the Heisenberg picture in Supplementary Fig. 1.

Originating from the Kirchhoff’s circuit laws, we express the boundary conditions for the annihilation operators as

(3) $$\frac{1}{\sqrt{Z_{0}}}({\hat{b}}_{\text{L}}-{\hat{b}}_{\text{R}})=-\frac{1}{\sqrt{R_{\text{A}}}}({\hat{a}}_{\text{R}}-{\hat{a}}_{\text{L}})$$

(4) $$\frac{1}{\sqrt{Z_{0}}}({\hat{c}}_{\text{R}}-{\hat{c}}_{\text{L}})=-\frac{1}{\sqrt{R_{\text{B}}}}({\hat{d}}_{\text{L}}-{\hat{d}}_{\text{R}})$$

(5) $$\sqrt{Z_{0}}({\hat{b}}_{\text{L}}+{\hat{b}}_{\text{R}})=\sqrt{R_{\text{A}}}({\hat{a}}_{\text{R}}+{\hat{a}}_{\text{L}})$$

(6) $$\sqrt{Z_{0}}({\hat{c}}_{\text{R}}+{\hat{c}}_{\text{L}})=\sqrt{R_{\text{B}}}({\hat{d}}_{\text{L}}+{\hat{d}}_{\text{R}})$$

(7) $${\hat{c}}_{\text{R}}=e^{i\phi }{\hat{b}}_{\text{R}}$$

(8) $${\hat{b}}_{\text{L}}=e^{i\phi }{\hat{c}}_{\text{L}}$$

where ϕ=ωs/v is the phase shift obtained by a wave with angular frequency *ω* and velocity *v* when travelling over distance *s*. Assuming no photons coming from the right, ${\hat{d}}_{\text{L}}=0$, we can solve the transmission coefficient *t* defined as ${\hat{d}}_{\text{R}}=t(\omega ){\hat{a}}_{\text{R}}.$ Thus, we obtain

(9) $$|t(\omega ){|}^{2}=\frac{2}{1+\frac{R_{\text{A}}^{2}+R_{\text{B}}^{2}}{2R_{\text{A}}R_{\text{B}}}+\frac{R_{\text{A}}^{2}R_{\text{B}}^{2}+Z_{0}^{4}-R_{\text{A}}^{2}Z_{0}^{2}-R_{\text{B}}^{2}Z_{0}^{2}}{2R_{\text{A}}R_{\text{B}}Z_{0}^{2}}\sin^{2}(\phi )}$$

The transmission coefficient is symmetric with respect to the exchange of resistances *R*_A_ and *R*_B_. In a matched case, *R*_A_ = *R*_B_ = *Z*_0_, equation (9) simplifies to |*t*(*ω*)|^2^ = 1.

Energy dissipation at the resistor *R*_B_ can be obtained from the average photon flux to the right in the transmission line with a characteristic impedance *R*_B_ multiplied by the energy carried by each photon. Here, the zero-point energy does not appear in the dissipated power. Thus, the power per unit frequency can be expressed as

(10) $$\begin{array}{l} P_{\to ,\omega }=\langle \hbar \omega {\hat{d}}_{\text{R}}^{\text{†}}{\hat{d}}_{\text{R}}\rangle \\ \;=\hbar \omega |t(\omega ){|}^{2}\langle {\hat{a}}_{\text{R}}^{\text{†}}{\hat{a}}_{\text{R}}\rangle \\ \;=\hbar \omega |t(\omega ){|}^{2}\frac{1}{\exp(\frac{\hbar \omega }{k_{B}T_{\text{A}}})-1} \end{array}$$

since the number of photons traveling right in Resistor A is given in thermal equilibrium by the Bose–Einstein distribution. Due to symmetry, the power transfer to the opposite direction is given by

(11) $$P_{\leftarrow ,\omega }=\hbar \omega |t(\omega ){|}^{2}\frac{1}{\exp(\frac{\hbar \omega }{k_{B}T_{B}})-1}$$

The net photonic heat transport from *R*_A_ to *R*_B_ is, therefore, given by

(12) $$\begin{array}{l} P_{\Gamma }=\int_{0}^{\infty }\frac{\text{d}\omega }{2\pi }(P_{\to ,\omega }-P_{\leftarrow ,\omega })\\ \;\;\;=\int_{0}^{\infty }\frac{\text{d}\omega }{2\pi }\hbar \omega |t(\omega ){|}^{2}\left[\frac{1}{\exp(\frac{\hbar \omega }{k_{B}T_{\text{A}}})-1}-\frac{1}{\exp(\frac{\hbar \omega }{k_{B}T_{\text{B}}})-1}\right] \end{array}$$

In the special case of a vanishing waveguide length, *s* → 0, equation (9) yields |*t*(*ω*)|^2^ = 4*R*_A_*R*_B_/(*R*_A_ + *R*_B_)^2^ which is identical to the result considered in ref. 30 for two resistors in a loop. On the other hand, if one sets *Z*_0_ to be inversely proportional to s and takes the limit *s* → 0, one obtains|*t*(*ω*)|^2^ = 4*R*_A_*R*_B_/[(*R*_A_ + *R*_B_)^2^ + *X*^2^] with a reactance *X* = *Z*_0_*ωs/υ*. This result reproduces that of two resistances connected in a loop with a series reactance^27^,^30^.

### NIS thermometry

The quasiparticle current through an NIS junction with tunnelling resistance *R*_T_ is given in the sequential-tunnelling theory by^7^

(13) $$I(V,T_{\text{N}})=\frac{1}{eR_{\text{T}}}\int_{0}^{\infty }n_{\text{S}}(E)[f(E-eV,T_{\text{N}})-f(E+eV,T_{\text{N}})]\text{d}E$$

where *T*_N_ is the normal-metal electron temperature, and *V* the voltage across the junction. Here, the Fermi-Dirac distribution is given by

(14) $$f(E,T)=\frac{1}{e^{E/(k_{\text{B}}T)}+1}$$

and the superconductor density of quasiparticle states assumes the form

(15) nS(E)=|ReE/Δ+iγ(E/Δ+iγ)2−1|

Above, γ is the Dynes parameter^7^ accounting for the subgap current, and Δ is the superconductor energy gap. Experimentally, *γ* is obtained as the ratio of the asymptotic resistance at large voltages and the resistance at zero voltage provided that we operate well below the critical temperature of the superconductor. We note that equation (13) has a very weak dependence on the temperature of the superconductor through the temperature dependence of Δ. Thus, an NIS junction can be used as a thermometer probing the electron temperature of the normal metal. We apply a constant current, and deduce the temperature from the measured voltage according to a calibration curve shown in Supplementary Fig. 2.

### Thermal model

In the full thermal model illustrated in Supplementary Fig. 2a, we consider several heat transfer mechanisms: Firstly, the NIS junctions produce heat flows between the normal-metal islands and the superconducting leads. Secondly, the electrons in the normal metal exchange heat with the phonon bath. Thirdly, the islands exchange heat with each other by photons travelling in the transmission line. Finally, the model takes into account geometrical properties of the samples as well as properties specific to the measurement setup.

**Figure 1:**
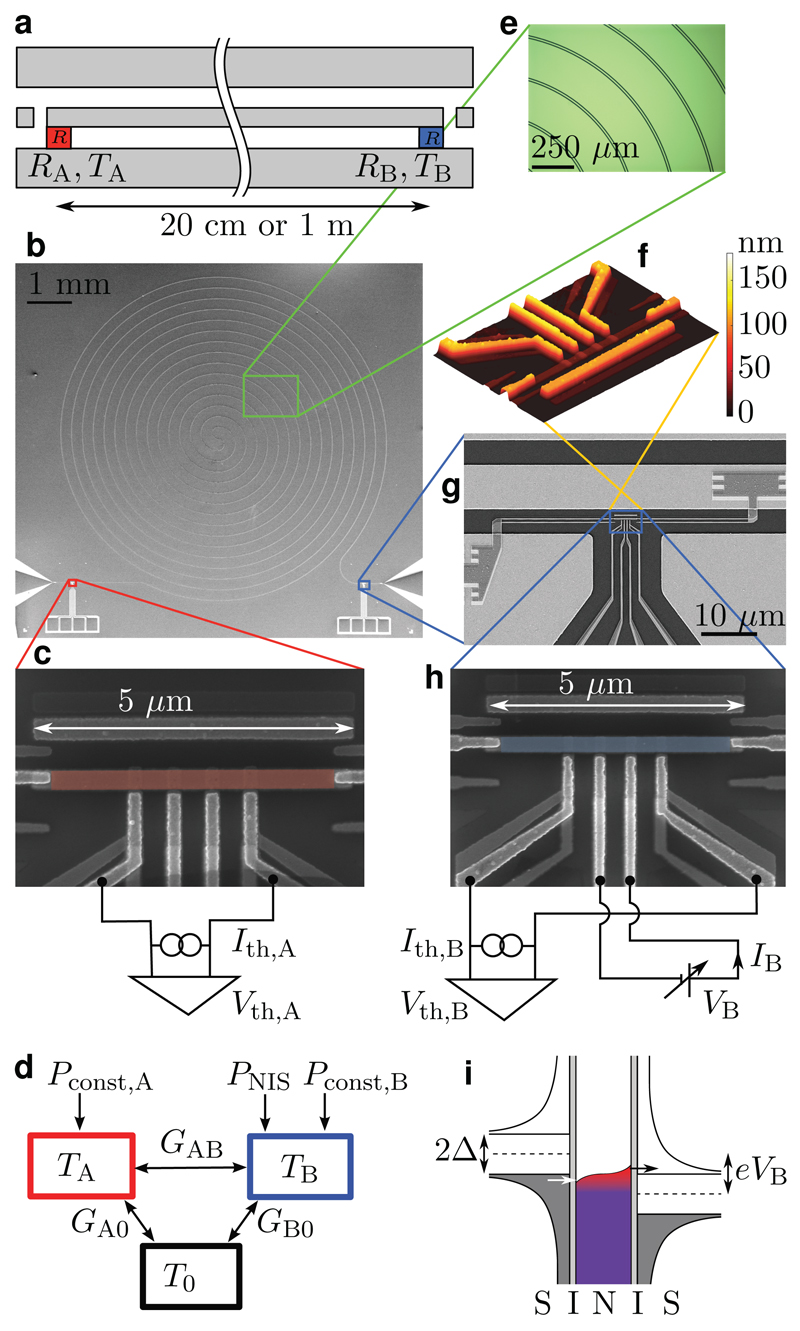
Sample structure and measurement scheme. **a**, Schematic illustration of a coplanar transmission line terminated at different ends by resistances *R*_A_ and *R*_B_ at electron temperatures *T*_A_ and *T*_B_, respectively. **b**, Scanning electron microscope (SEM) image of a fabricated transmission line with a double-spiral structure. **c, h**, False-colour SEM images of the normal-metal islands together with a simplified measurement scheme. **e**, Optical micrograph of the waveguide. **f**, Atomic force microscope image of Island B highlighting the thicknesses of the nanostructures. **g**, SEM image showing how the normal-metal island is connected to the ground plane and to the centre conductor. Micrographs (**c, f, g, h**) are from Sample A1, and (**b, e**) are from a similar sample. **h**, Thermal model indicating the thermal conductance between the Islands A and B, *G*_AB_, and those from the islands to the phonon bath at the temperature *T*_0_, *G*_A0_ and *G*_B0_. Constant powers *P*_const,A/B_ and control power *P*_NIS_ are also indicated by arrows. **i**, Schematic diagram for cooling of the normal metal due to single-electron tunnelling (arrows) in a pair of NIS junctions biased at voltage $V_{\text{B}}≲2\Delta /e$. The densities of states in the superconductors (S) are shown by black solid lines whereas the Fermi distribution is indicated in the normal metal (N).

The NIS junctions can be used for cooling^7^, ^31^ (Fig. 1i) and heating of the normal metal. The power out of the normal metal can be computed from^7^

(16) $$P_{\text{ideal}}=\frac{1}{e^{2}R_{\text{T}}}\int_{-\infty }^{\infty }n_{\text{S}}(E)(E-eV)[f(E-eV,T_{\text{N}})-f(E,T_{\text{s}})]\text{d}E$$

We model the nonidealities in the NIS power by assuming a constant fraction, *β*, of the power flowing to the superconductor to flow back to the normal metal. Thus, the back-flow power can be written as

(17) $$P_{\text{bf}}=\beta (IV+P_{\text{ideal}})$$

where *IV* gives the total power. Consequently, the total cooling power of an NIS junction is given by

(18) $$P_{\text{NIS}}=P_{\text{ideal}}-P_{\text{bf}}$$

The physical background for the back flow has been studied in ref. 32. A factor of 2 is included in the power when two NIS junctions are connected to form an SINIS structure. Since the thermometers are based on similar NIS junctions, their powers are calculated with the same equations as for the actual power used to control the temperature of Island B. However, the voltages across the thermometer junctions must first be solved using equation (13), the island temperature, and the thermometer bias current.

The electrons in the normal metal are coupled to the phonon bath, and the heat flow is given by^7^

(19) $$P_{\text{ep},i}=\Sigma {}_{\text{N}}\Omega_{i}(T_{i}^{5}-T_{0}^{5})$$

Here, Ω_*i*_ is the volume of normal-metal block *i* є {A, B, AR, BR}. For Cu and AuPd, the parameter Σ_N_ is typically^7^, ^13^ between 2 × 10^9^ and 4 × 10^9^ WK^−5^m^−3^. In the simulations, we use values 2.0 × 10^9^ WK^−5^m^−3^ and 3.0 × 10^9^ WK^−5^m^−3^ for Cu and AuPd, respectively, unless otherwise mentioned. The normal metal under the superconductors at the ends of the islands are excluded from the volume in the simulations due to the superconductor proximity effect. For small temperature differences, *T_i_* ≈ *T*_0_, one obtains

(20) $$P_{\text{ep},i}=G_{\text{ep},i}(T_{i}-T_{0})$$

where $G_{\text{ep},i}=5\Sigma_{\text{N}}\Omega_{i}T_{0}^{4}$.

We account for heat leaks from a high-temperature environment by including constant heating powers to both islands, P_leak,A_ and P_leak,B_. They are fixed by the saturation of the electron temperature observed in Supplementary Fig. 2 at low bath temperatures.

In the thermal model, we consider quasiparticle heat conduction only from the islands to their near-by normal-metal reservoirs. The reservoirs are a consequence of the three-angle evaporation method, and they provide an additional channel for thermalization to the phonon bath. At both islands, there are actually two reservoirs which are presented as one in Supplementary Fig. 2a for simplicity. The extremely weak quasiparticle heat conduction from one island to the other over a distance longer than 5 mm is included in the parasitic heat conduction as discussed below. The power flow at the normal-metal block *i* due to the quasiparticles is given by^13^

(21) $$P_{\text{qp},i}=\kappa_{\text{S}}AT^{\prime }(x_{i})$$

where *T*′(*x_i_*) is the derivative of the quasiparticle temperature in the superconductor with respect to the position coordinate *x_i_*, and *A* is the cross section of the line. The superconductor heat conductivity, *κ*_S_, is related to the normal-state heat conductivity, *κ*_N_, at a temperature *T* by^33^

(22) $$\kappa_{\text{S}}=\tilde{\gamma }\text{(}T\text{)}\kappa_{\text{N}}$$

where $\tilde{\gamma }$ is a suppression factor

(23) $$\tilde{\gamma }\text{(}T\text{)}=\frac{3}{2\pi^{2}}\int_{\Delta /(\kappa_{\text{B}}T)}^{\infty }\frac{t^{2}}{\cosh^{2}(t/2)}\text{d}t$$

The normal-state heat conductivity of the line is obtained from the Wiedemann-Franz law as

(24) $$\kappa_{\text{N}}=\frac{L_{0}T(x)}{\rho }$$

where *ρ* is the normal-state electric resistivity of the line, and *L*_0_ = 2.4 × 10^−8^ WΩK^2^ is the Lorenz number. The temperature profile in the superconducting lines can be calculated using a heat diffusion equation^13^. However, the electron–phonon coupling in a superconducting state is greatly suppressed with respect to that of a normal-state^34^. Thus, we neglect the electron–phonon coupling in the leads and assume here a linear temperature profile.

Andreev current plays a minor role in our experiments since the induced temperature changes at the islands are small in the subgap voltage regime where it may dominate^35^. Therefore, we do not consider it in the thermal model.

We observe a weak island-to-island heat transport also in the control sample, in which the centre conductor is shunted as shown in Supplementary Fig. 3. We model this parasitic heat transport by letting a constant proportion, *α*, of the total input power at Island B to flow into Island A,

(25) $$P_{\text{p}}=\alpha I_{\text{B}}V_{\text{B}}$$

The exact mechanism of the parasitic channel remains unknown, and the heat flow may depend on the sample geometry. The parasitic heat conduction extracted from the control sample includes all the heat conduction channels from one island to the other except the photonic heat conduction which is essentially absent due to the shunt. This heat flow may be attributed to quasiparticles since they can travel long distances before recombination, especially at low bath temperatures. Furthermore, although the electric contact of the shunting metal block between the ground plane and the centre conductor is of very low impedance, small residual photonic heat conduction cannot be fully excluded. Nevertheless, the parasitic heat conduction is much weaker than the total heat conduction in the actual devices. We note that the parasitic heat conduction is only added to the model for more accurate description at high heating powers.

We solve the heat balance equations for both islands and both reservoirs simultaneously. The equations can be expressed as (Supplementary Fig. 2a)

(26) $$P_{\Gamma }+P_{\text{th,A}}-P_{\text{leak,A}}-P_{\text{p}}+P_{\text{ep,A}}+P_{\text{qp,A}}=0$$

(27) $$P_{\text{NIS}}-P_{\Gamma }+P_{\text{th,B}}-P_{\text{leak,B}}+P_{\text{p}}+P_{\text{ep,B}}+P_{\text{qp,B}}=0$$

(28) $$P_{\text{ep,AR}}-P_{\text{qp,A}}=0$$

(29) $$P_{\text{ep,BR}}-P_{\text{qp,B}}=0$$

These equations yield the temperatures *T_i_*, *i* є {A, B, AR, BR} for a given phonon bath temperature, *T*_0_, and bias voltage, *V*_B_, both of which are accurately controlled.

The parameters used in the full thermal model are shown in Supplementary Table 1. In the simulations, we slightly adjust the quasiparticle heat conductivity for improved agreement between the model and the experiments. More specifically, we set the temperature in equation (23) to be equal to the island temperatures increased by a small constant value and, in addition, we set the suppression factor to saturate at low temperatures. Hence, we introduce a replacement $\tilde{\gamma }(T)\to \tilde{\gamma }(T+T_{\text{const}})+\tilde{\gamma }(T_{\text{satur}})$. This approximation can be justified by several arguments. Firstly, the superconductor heat conductivity depends on the purity of the sample^36^. Secondly, the superconductor energy gap has been observed to increase at small film thicknesses^37^. We use for all superconductors the same value, which is obtained from the current–voltage measurements of the NIS junctions, although the leads are thicker. The possibly smaller actual energy gap effectively corresponds to higher temperatures. Thirdly, the neglected electron–phonon coupling in the superconducting leads may result in nonlinear temperature profile increasing the quasiparticle heat conduction. The impurities in the sample may increase the electron–phonon coupling. Fourthly, the heat leakage through the measurement cables from a high-temperature environment and other possible heat leak mechanisms may increase the temperature of the superconductors. Increased quasiparticle densities have been observed previously, and they can be suppressed by effective shielding and enhanced relaxation^38^. Weak quasiparticle recombination can induce elevated quasiparticle temperatures. However, we increase only the heat conductivity and consider a linear temperature profile in the lead between the island and the near-by reservoir. In the simulations, the reservoirs have effective volumes somewhat larger than their physical volumes, thus, taking into account the quasiparticles thermalizing in the reservoirs and the ones recombining in the superconductors. The requirement of the effective volume may also be explained by the uncertainty in the employed literature value of the electron–phonon coupling constant^7^.

### Additional control samples without resistors

We also fabricated and measured control samples without the normal-metal resistors terminating the transmission line. Instead, the transmission line is connected to input and output ports through coupling capacitors forming a resonator. The ground planes at both sides of the centre conductor are connected by bond wires to suppress possible slot line modes in these additional control samples as well as in the actual samples.

Supplementary Figure 4 shows a measured transmission coefficient *S*_21_ of the resonator as a function of frequency. The resonance peaks are located at the design positions and they exhibit much higher transmission than the other visible features. The PCB and other parts of the system cause some apparent resonances in the figure. From the *S*_21_ parameter, one can extract the loaded quality factor, which depends on the internal losses and the external losses through the coupling capacitors. Using an LCR model^39^, we estimate that the internal quality factor of the system can reach values of the order of 60,000 indicating a negligibly weak effect in the heat conduction experiments. In fact, the energy losses due to the observed finite quality factor would only limit the photonic heat conduction beyond distances of the order of a kilometre. For our measurements, internal quality factors of the order of 100 would be sufficient.

The characteristic impedance of the coplanar waveguide is designed to be approximately 50 Ω, and this design value agrees well with the experiments. Possible deviations of the order 10% from the design value change the photonic heat conduction on the single percent level.

## Supplementary Material

Supplementary information

## Figures and Tables

**Figure 2:**
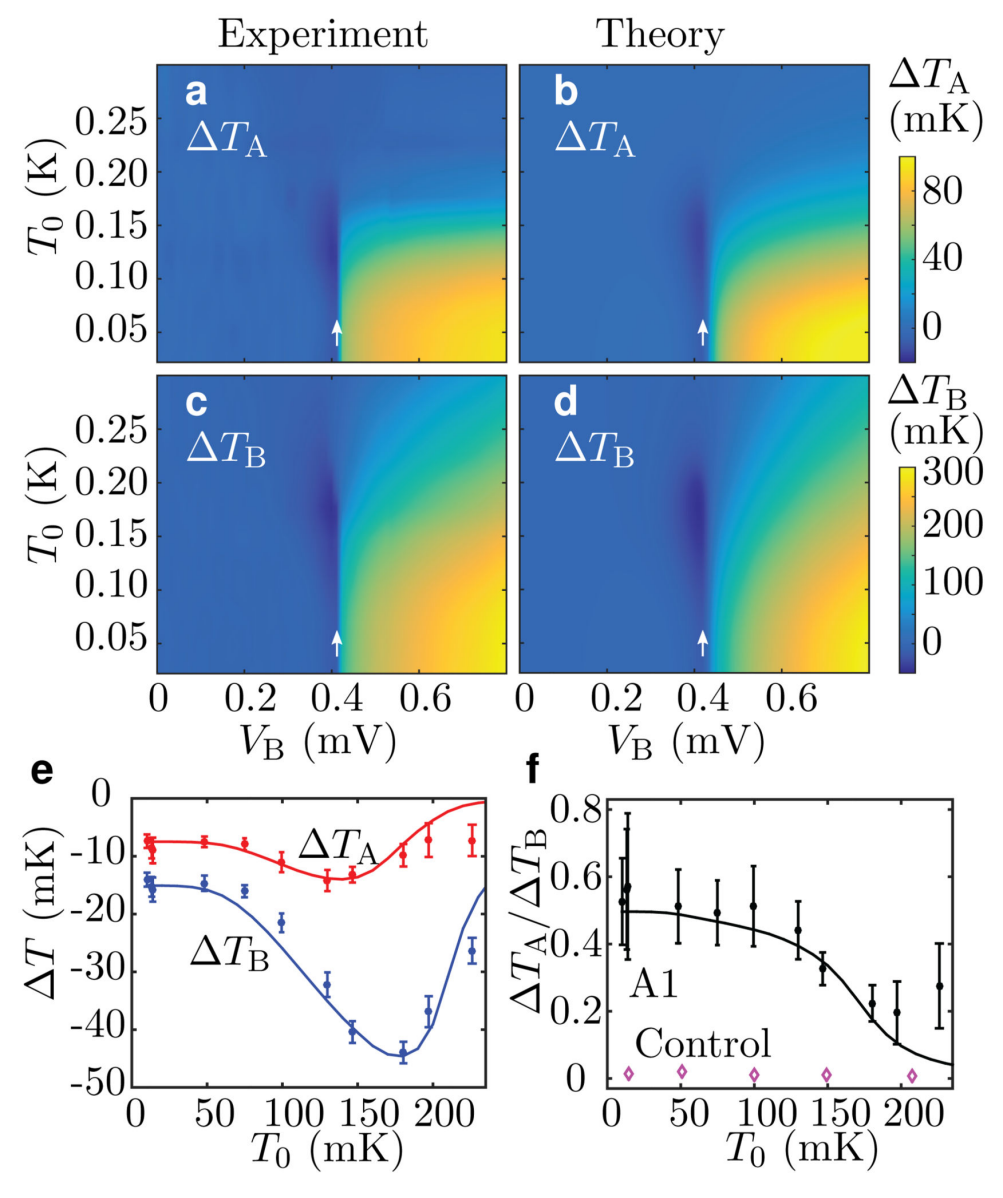
Photonic cooling at macroscopic distances for Sample A1. **a, b**, Measured (**a**) and theoretically predicted (**b**) electron temperature changes with respect to the zero-bias case (*V*_B_ = 0) for Island A as functions of the voltage *V*_B_ and bath temperature *T*_0_. At each *T*_0_, the maximum cooling is obtained at *V*_B_ ≈ 0.4 mV ≈ 2Δ/*e*, as indicated by the white arrows. **c, d**, As in panels (**a, b**), but for temperature changes of Island B. **e**, Measured (markers) and simulated (lines) temperature changes at the maximum cooling point as functions of the bath temperature. The errorbars indicate the standard deviation of the measured temperatures. **f**, The ratio of the temperature changes in (**e**). Measurement results from the control sample (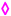) are shown for comparison.

**Figure 3:**
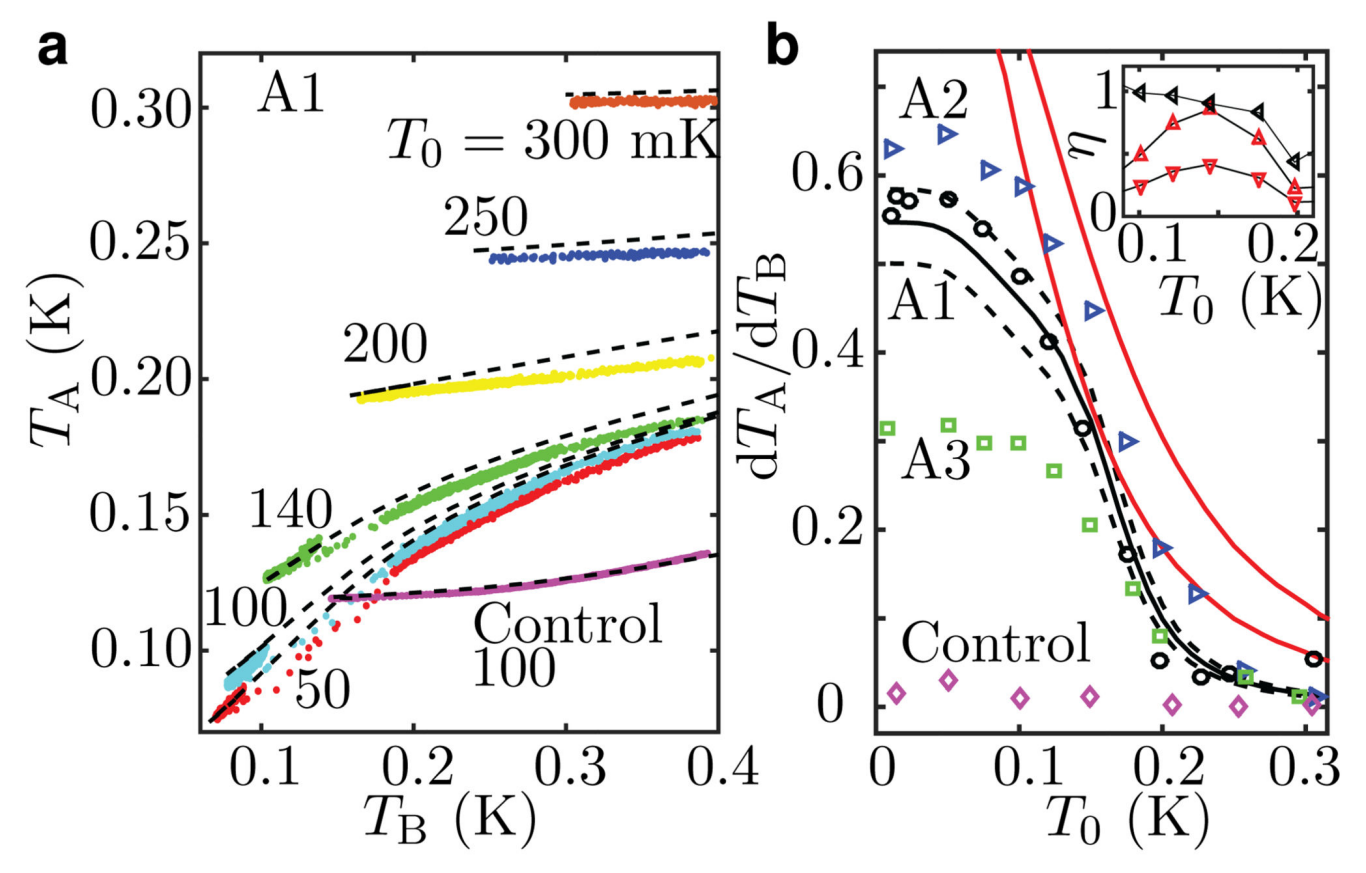
Differential temperature response and the quantum of thermal conductance. **a**, Measured (dots) and simulated (dashed lines) temperatures of Island A as functions of the temperature of Island B for the indicated phonon bath temperatures in Sample A1. The results for the control sample at 100-mK bath temperature are shown for comparison. **b**, Differential temperature response (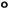) from (**a**) at the lowest *T*_B_ for each bath temperature. The experimental uncertainty is of the order of the marker size. For comparison, we also show the corresponding experimental data for the control sample (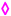), for Sample A2 (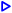), and for A3 (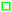). The solid black line shows the prediction of the full thermal model of Supplementary Fig. 2. The dashed lines are calculated with 80 % (bottom) and 115 % (top) of the quantum of thermal conductance indicating the sensitivity of the results to the photonic heat conduction. The solid red lines are calculated with the simplified thermal model (equation (2)) for Sample A1 with electron–phonon coupling constants^7^ Σ_N↓_ = 2 × 10^9^ WK^−5^m^−3^ (right), and Σ_N↑_ = 4 × 10^9^ WK^−5^m^−3^ (left). The inset shows the extracted fraction *η* = *G*_AB_/*G*_Q_ for Sample A1 for the simplified model with Σ_N↓_ (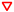) and Σ_N↑_ (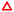) and for the full thermal model (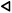) as functions of *T*_0_.

**Table 1:** Main parameters of the measured samples. Columns show the waveguide lengths, normal-metal resistances *R_i_*, *i* є {A, B}, normal-metal materials, and normal-metal volumes (length×width×thickness). Based on the resistance values and equation (1), the right-most column provides the estimated ratio of the realized photonic thermal conductance and the quantum of thermal conductance at temperatures of approximately 150 mK.

Sample	Length (m)	*R_i_* (Ω)	Material	Volume (nm^3^)	*G*_Γ_/*G*_Q_
A1	0.2	65	Cu	5000 × 300 × 20	98 %
A2	1.0	75	AuPd	3200 × 300 × 40	94 %
A3	0.2	150	AuPd	3200 × 300 × 20	60 %
Control	0.2	100	AuPd	3200 × 300 × 20	0 %
