# Supplementary material for: Quantum-limited heat conduction over macroscopic distances
Source: Nat Phys. Author manuscript; Available in PMC 2016 Sep 22. (PMC4878655; doi:10.1038/nphys3642)
Supplement: Supplementary information [file NIHMS66521-supplement-Supplementary_information.pdf]

Supplementary information

Quantum-limited heat conduction over macroscopic distances

Matti Partanen, Kuan Yen Tan, Joonas Govenius, Russell E. Lake, Miika K. Mäkelä, Tuomo Tanttu, and Mikko Möttönen

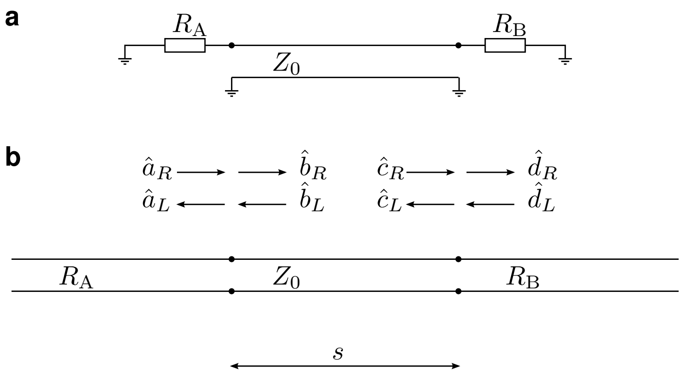

Supplementery Figure 1: **Transmission line diagrams.** **a**, Circuit diagram of a transmission line with characteristic impedance Z_0 terminated by resistors $R_{\rm A}$ and $R_{\rm B}$. **b**, System in (**a**) represented as three connected transmission lines. The annihilation operators for the left- and right-moving photons at the different points of the system are denoted by $\hat{a}_i, \hat{b}_i, \hat{c}_i$, and $\hat{d}_i$, $i \in \{\text{L,R}\}$.

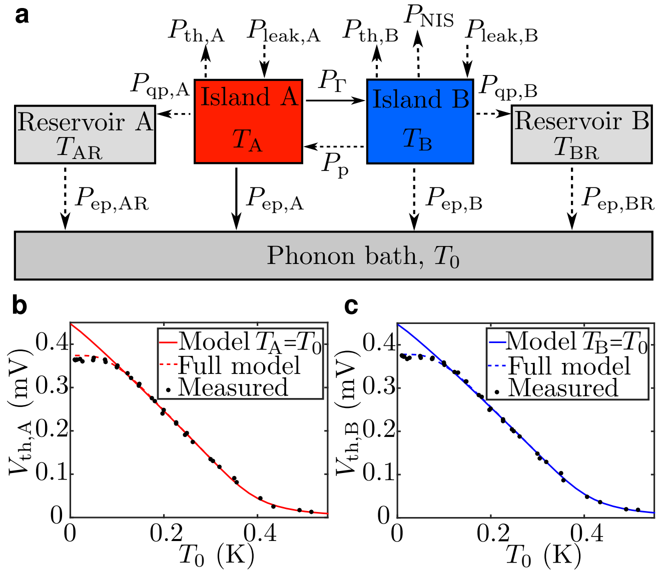

Supplementery Figure 2: **Thermal model and temperature calibration. a**, In the full thermal model, the conduction electron systems of the normal-metal islands exchange heat with each other by photonic heat conduction, P_Γ, and with the near-by normal-metal reservoirs through quasiparticle heat conduction, $P_{{\rm qp},i}$. The electrons exchange heat directly with the phonon bath through the electron–phonon coupling $P_{{\rm ep},i}$. The temperature of Island B is controlled with power of the NIS junction, $P_{\rm NIS}$, and the thermometers introduce powers $P_{{\rm th},i}$. Heat leakages from a high-temperature environment, $P_{{\rm leak},i}$, are also included in the model as well as a parasitic heat flow between the islands, $P_{\rm p}$. Here, subscripts A, B, AR, and BR denote Islands A and B, and the corresponding reservoirs, respectively. The simplified model accounts only for P_Γ and $P_{\rm ep,A}$, which are denoted by solid arrows. See Methods for details. **b, c**, Thermometer voltages as defined in Figs. 1c, h in the main article for Island A (**b**) and B (**c**) as functions of the bath temperature in Sample A1. The solid lines show theoretical predictions without any free parameters assuming that the electron temperature is equal to the bath temperature. These curves are used for the voltage–temperature conversion. The discrepancy between the data and the model below 100 mK indicates the saturation of the electron temperature, confirmed by the dashed lines showing the theoretically predicted voltages with the electron temperature solved from the full thermal model. The uncertainty in the measurements is of the order of the marker size.

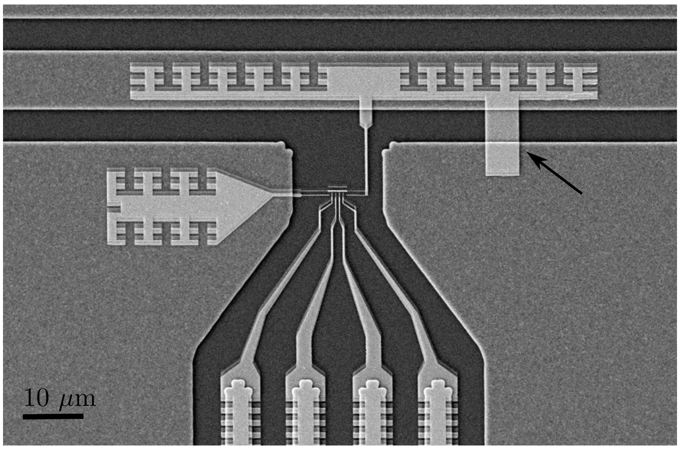

Supplementery Figure 3: **Control sample.** SEM image of the control sample. The centre conductor of the coplanar waveguide is shunted to the ground plane at the location indicated by the black arrow.

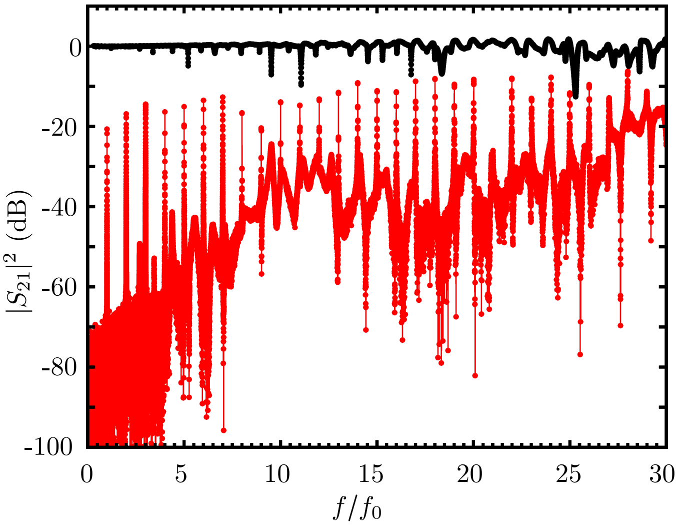

Supplementery Figure 4: **Resonance measurement.** Measured transmission coefficient of a 20-cm long resonator (red dots) as a function of frequency normalized with the fundamental frequency $f_0 = 330$ MHz. Lines are guides for the eye. The resonator is terminated at both ends by 2+2 interdigital coupling capacitors with a finger length of 100 μm, and finger width and separation of 5 μm. The loaded quality factor of the first resonance peak is 4.4×10^4. We also measured a sample without coupling capacitors (black), i.e., a continuous transmission line with otherwise identical structure. The transmission coefficients are normalized with the approximate attenuation in the connectors and cables outside the sample. The resonator and the transmission line are fabricated out of Nb and they are measured at 4.2 K.

Supplementery Table 1: **Simulation parameters for Sample A1.** Sample dimensions are based on the actual sample, $I_{\mathrm{th,A}}$ and $I_{\mathrm{th,B}}$ are externally controlled, Δ, γ and resistances are extracted from independent current–voltage characteristics, ρ assumes a typical value for evaporated Al, L_l is calculated analytically[1,2], C_l is based on a finite-element simulation, $P_{\mathrm{leak,A}}$ and $P_{\mathrm{leak,B}}$ are obtained from island temperature saturation at low phonon bath temperatures in Supplementary Fig. 2, α is extracted from the control sample, β is obtained from the nonideal cooling power of the NIS junctions, Ω_{AR} and Ω_{BR} are the actual volumes multiplied by 2 to account also for the quasiparticle recombination in the superconductors, and T_{satur} and T_{const} are fitting parameters assuming realistic values.

Parameter	Symbol	Value	Unit
Island volume	Ω_{A}, Ω_{B}	$4500 \times 300 \times 20$	nm^3
Effective reservoir volume	$\Omega_{\mathrm{AR}}, \Omega_{\mathrm{BR}}$	$10 \times \Omega_{\mathrm{A}}$	
Cross section of lines from island to reservoir	A	300×100	nm^2
Distance from island to reservoir	l_{IR}	24	μm
Waveguide length	s	0.193	m
Superconductor energy gap	Δ	224	μeV
Inductance per unit length	L_l	4.14×10^{-7}	H/m
Capacitance per unit length	C_l	1.51×10^{-10}	F/m
Thermometer bias current	$I_{\mathrm{th,A}}$, $I_{\mathrm{th,B}}$	18	pA
Resistivity of Al lines in normal state	ρ	1.0×10^{-8}	Ωm
Dynes parameter	γ	1.05×10^{-4}	
Material parameter for Cu	Σ_{N}	2.0×10^{9}	W K^{-5} m^{-3}
Normal state junction resistance	R_{T}	15.5	kΩ
Island resistance	R_{A}, R_{B}	65	Ω
Characteristic impedance	Z_0	$\sqrt{L_l/C_l}$	
Phase velocity	v	$1/\sqrt{C_l L_l}$	
Lorenz number	L_0	2.4×10^{-8}	W Ω K^{-2}
Quasiparticle saturation temperature	T_{satur}	0.184	K
Addition to quasiparticle temperature	T_{const}	0.036	K
Parasitic heat conduction parameter	α	3×10^{-4}	
NIS back flow constant	β	0.056	
Heat leak	$P_{\mathrm{leak,A}}$, $P_{\mathrm{leak,B}}$	1.6	fW

References

[1] S. Gevorgian, L.J.P. Linner, and E.L. Kollberg. CAD models for shielded multilayered CPW. *Microwave Theory and Techniques, IEEE Transactions on*, 43(4):772–779, Apr 1995.

[2] M. Göppl, A. Fragner, M. Baur, R. Bianchetti, S. Filipp, J. M. Fink, P. J. Leek, G. Puebla, L. Steffen, and A. Wallraff. Coplanar waveguide resonators for circuit quantum electrodynamics. *J. Appl. Phys.*, 104(11), 2008.
